# Histopathologische Befunde bei therapierter und nichttherapierter SARS-CoV-2-Infektion – Bericht über 3 Autopsien

**DOI:** 10.1007/s00194-020-00408-x

**Published:** 2020-07-06

**Authors:** R. Dettmeyer, G. Lasczkowski, A. Weber, T. Wolter, G. Kernbach-Wighton

**Affiliations:** Institut für Rechtsmedizin, Justus-Liebig-Universität Gießen, Frankfurter Str. 58, 35392 Gießen, Deutschland

**Keywords:** COVID-19, Histopathologie, Lunge, Leber, Niere, Myokard, COVID-19, Histopathology, Lungs, Liver, Kidneys, Myocardium

## Abstract

Bei letalem Verlauf einer SARS-CoV-2-Infektion kommt nach bisherigem Kenntnisstand eine Beteiligung mehrerer innerer Organe in Betracht. Im Vordergrund stehen pathologische Befunde im Lungengewebe, berichtet wird aber auch von direkt oder indirekt als Folge einer Infektion mit SARS-CoV‑2 auftretenden (histo-)pathologischen Befunden im Nierengewebe, in der Leber und im Myokard. Der Vergleich der histopathologischen Diagnostik mit konventionell-histologischen Färbungen bei 3 im Zusammenhang mit einer SARS-CoV-2-Infektion verstorbenen Männern zeigt teils identische Befunde und erlaubt Überlegungen zu Chronologie und Pathophysiologie des Krankheitsverlaufes. Zwei Männer wurden intensivmedizinisch invasiv beatmet; ein Mann starb nach 8 Tagen häuslicher Quarantäne ohne Therapie. Es zeigt sich ein großes Spektrum SARS-CoV-2-assoziierter Befunde.

## Abstract (en)

According to the current state of knowledge, several internal organs are usually involved in cases of SARS-CoV‑2 infections with a fatal course. Pathological changes are primarily found in lung tissues but there are also reports concerning direct or indirect (histo)pathological changes due to SARS-CoV‑2 infections in samples from the kidneys, liver and myocardium. Comparing three fatal cases associated with SARS-CoV‑2 infections in men using conventional histological staining, there were partly identical findings that enabled interpretations with respect to the chronology and pathophysiology of the disease. Of the men two were invasively ventilated in the intensive care unit and one man died after 8 days in domestic quarantine without treatment. A wide spectrum of findings potentially associated with SARS-CoV‑2 must be taken into account.

## Einleitung

Die Pandemie mit SARS-CoV‑2 führt in Relation zur Zahl der bekannten Infizierten zu einer beachtlichen Mortalität, insbesondere Risikogruppen betreffend. Genannt werden als Risikofaktoren das Alter (>60 Jahre), das Geschlecht (männlich) und Begleiterkrankungen, v. a. Diabetes mellitus, eine COPD, Adipositas und rheumatische Erkrankungen. Klinisch verläuft die Infektion in der Anfangsphase weniger gravierend, bei letalen Verläufen wird jedoch von einer rasch auftretenden Verschlechterung des Zustands berichtet. Die Patienten werden intensivpflichtig und müssen, zunächst nichtinvasiv, später jedoch mitunter invasiv beatmet werden. Radiologische Befunde demonstrieren die ausgedehnte Schädigung des Lungengewebes mit „milchglasartigen“ Verschattungen, die für eine SARS-CoV-2-Infektion charakteristisch sein sollen [14]. Berichtet wird von histologischen Befunden, wie einer möglicherweise viruscharakteristischen Endotheliitis, sowie von einer erhöhten Thromboseneigung [10, 15, 18]. Im Nierengewebe sollen inflammatorische Prozesse im Sinne einer Nephritis auftreten können. Berichtet wird zudem von einer „collapsing glomerulopathy“ [6, 11]. Auch eine COVID-19-assoziierte Myokarditis ist möglich. Wenn zunächst auch Strömungen gegen Obduktionen bei SARS-CoV-2-Verstorbenen bestanden, so ist doch zu konstatieren, dass die Obduktion gerade in Zeiten neuer und damit noch nicht hinreichend sicher zu beurteilender Herausforderungen zum einen zur Klärung der Todesursache beiträgt und zum anderen über pathomorphologische Befunde klinische Beobachtungen zum Krankheitsverlauf begreifbar macht. So können Obduktionen Einfluss auf das Behandlungsmanagement haben und sind nicht zuletzt eine qualitätssichernde Maßnahme. Daneben sind Aspekte der Gewebegewinnung für Forschungszwecke zu erwähnen. Obduktionen in der Rechtsmedizin sowie in der Pathologie tragen zur weiteren Aufklärung bei, ihre Ergebnisse sollen einer suffizienten Therapie von Patienten zugutekommen [2–6, 8–10, 13].

## Fallberichte

Bei 3 im Zusammenhang mit einer SARS-CoV-2-Infektion verstorbenen Männern konnten unter Aussparung des Zentralnervensystems im Nachgang zu einer Obduktion mit konventionell-histologischen Färbungen histopathologische Befunde erhoben werden. Die Männer waren zwischen 59 und 67 Jahre alt. In 2 Fällen war eine intensivmedizinische Behandlung mit längerer invasiver Beatmung vorangegangen. Der 3. Mann wurde 8 Tage nach positiver Testung auf SARS-CoV‑2 und häuslicher Quarantäne ohne Therapie leblos in seiner Wohnung gefunden. Bei 2 Patienten war ein Diabetes mellitus bekannt, bei dem 3. Patienten eine Cholezystolithiasis. Alle Männer hatten einen BMI über 27. Zwei Patienten waren in der Klinik zuletzt SARS-CoV-2-negativ getestet worden.

## Obduktionsergebnisse

Makroskopisch dominierte in allen 3 Fällen ein ödematöses, palpatorisch partiell verfestigtes, jedoch nur in einem Fall fokal etwas brüchiges Lungengewebe mit grau-rot-braun marmorierter Schnittfläche ohne deutlich abgrenzbare fokale makroskopische Veränderungen. In einem Fall war in den zentralen und peripheren Ästen des Bronchialbaums muzinös-grau-grüner Inhalt anzutreffen, in den beiden anderen Fällen stellten sich die oberen Atemwege frei durchgängig dar, in der Peripherie mit Bronchiektasen und ein schaumiges partiell hämorrhagisches Lungenödem. Die hilären Lymphknoten zeigten sich jeweils nur leicht prominent. Alle Verstorbenen zeigten eine Kardiomegalie und eine altersentsprechende, jedoch nicht höhergradig stenosierende Koronarsklerose, kleine graue narbige Areale in den Spitzen der Stellmuskeln, ein abgeflachtes Trabekelsystem und fokale Endokardfibrosen. Einmal zeigte sich das Myokard ebenso wie die Skelettmuskulatur etwas mürbe erweicht, das Lebergewebe jeweils etwas teigig aufgehellt, teilweise eher ockerfarben, in einem Fall gering ikterisch. Makroskopisch wiesen die Nieren altersentsprechende Befunde auf, mit leichter Verschmälerung der Nierenrinden und einer geringen bis mäßiggradigen Arterio-Arteriolosklerose. Die Organgewichte und die makroskopischen Organbefunde der Lungen sind in Tab. 1 gelistet.

**Table 1:** 

	Nr. 1 – therapiert	Nr. 2 – unbehandelt	Nr. 3 – therapiert
Lunge links	874 g	1352 g	618 g
Lunge rechts	1002 g	1568 g	752 g
Lungengewebe	Grau-glasig marmoriert, z. T. karnifiziert, z. T. brüchig	Marmoriert, grau-rot-braun, nicht eindeutig brüchig	Zum Teil karnifiziert, nichtbrüchig
Gehirn	1490 g	1464 g	1442 g
Niere links	294 g	196 g	266 g
Niere rechts	276 g	212 g	224 g
Herz	572 g	466 g	756 g
Leber	2188 g	1770 g	2322 g
Thromben	Nein	Nein	Ja

Bei den Obduktionen konnte makroskopisch weiterhin eine Dilatation der Herzhöhlen, insbesondere des rechten Herzvorhofs und der rechten Herzkammer gesehen werden, in 2 Fällen keine Lungenthromboembolie, in einem Fall sehr kleine Thromben in einzelnen peripheren Pulmonalarterienästen, insgesamt keine tiefen Beinvenenthrombosen.

## Histologie

Bei makroskopisch altersentsprechenden Befunden des Ösophagus, des Gastrointestinaltrakts und der ableitenden Harnwege wurden von diesen Lokalisationen keine histologischen Schnitte angefertigt.

Vom Lungengewebe wurden aus jedem Lungenlappen Proben aus der Peripherie und aus hilusnahen zentralen Arealen mikroskopisch untersucht, ebenso Lungenhiluslymphknoten, jeweils zahlreiche Proben vom Myokard und aus der Leber, zudem Proben von den Nieren, dem Pankreas, der Prostata, den Nebennieren und der Schilddrüse.

Die mikroskopischen Untersuchungen mit konventionell-histologischen Färbungen (Hämalaun-Eosin, Elastica-van-Gieson, PAS, Grocott, Berliner Blau) ergaben bei den 3 Fällen keine signifikanten oder keinesfalls auf eine SARS-CoV-2-Infektion zu beziehende Befunde im Pankreas, in den Nebennieren und in der Prostata.

### Histopathologie des Lungengewebes

**Figure 1:**
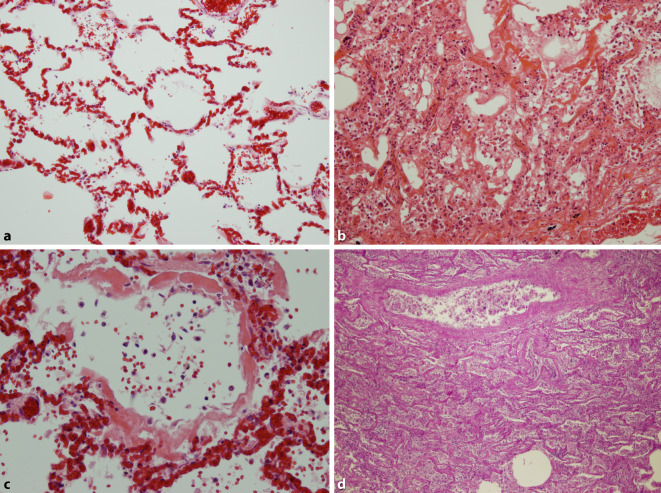

**Figure 2:**
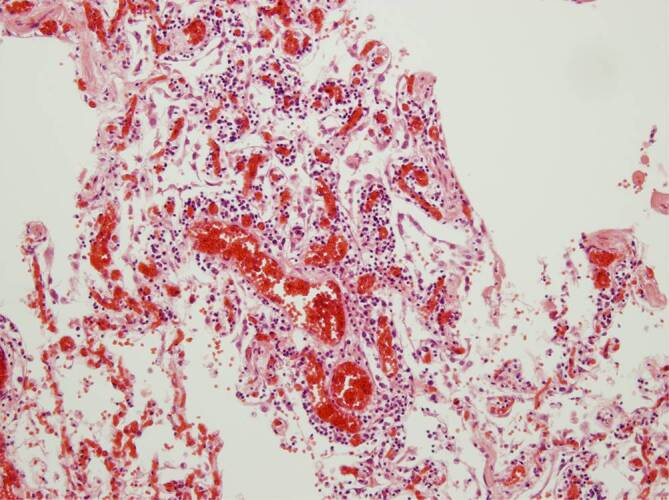

**Figure 3:**
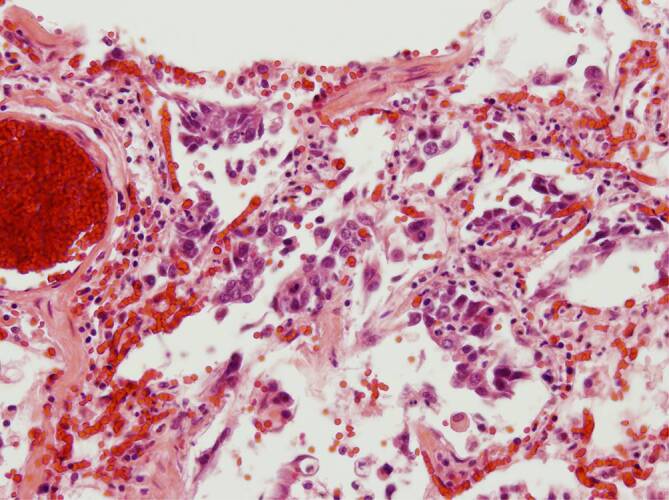

Im Lungengewebe zeigten sich regulär entfaltete und belüftete Areale mit akuter Stauungshyperämie unter Einbeziehung der Septumkapillaren (Abb. 1a). In anderen Regionen stellten sich ausgeweitete Lungenalveolen dar, mit interstitieller, lymphozytärer Entzündung und Alveolitis (Abb. 1b). Die Lichtungen der Lungenalveolen waren membranartig von homogen-eosinophilen Belägen geradezu austapeziert (Abb. 1c; Fall 2), das Lungengewebe häufig mit in Organisation begriffenen hyalinen Membranen, entsprechend einer Karnifizierung alteriert (Abb. 1d, Fall 1). Dazu fanden sich eine erhebliche Dilatation der Septumkapillaren und abgeschilferte Alveolarmakrophagen bei unterschiedlich ausgeprägter lymphozytärer Infiltration entlang der Kapillaren (Abb. 2), gelegentlich auch perivaskulär, selten mit diskreter Ausdehnung bis an das Gefäßendothel. Die Alveolarmakrophagen, aber offenbar auch das respiratorische Epithel peripherer Äste des Bronchialbaums, wiesen eine auffällige Zell- und Kernpolymorphie auf, häufig mit prominenten Nukleoli in den Zellkernen, die leicht balloniert wirken – insgesamt im Sinne einer gering- bis mäßiggradigen Dysplasie (Abb. 3).

### Histopathologie des Lebergewebes

**Figure 4:**
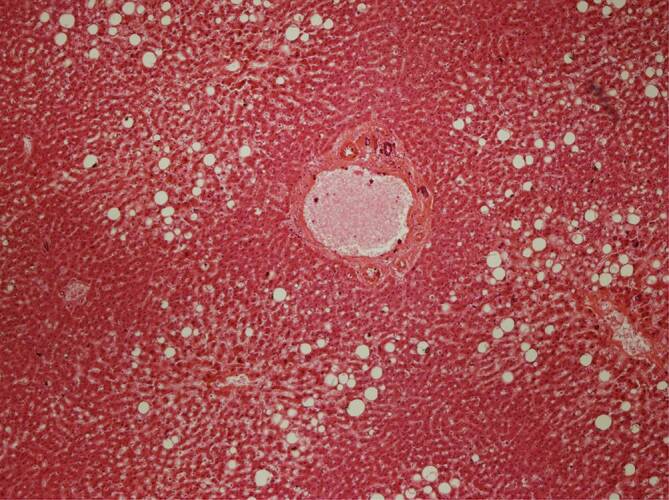

Das bereits partiell autolytische Lebergewebe wies in allen 3 Fällen eine unterschiedlich ausgeprägte Portalfibrose auf, in einem Fall einen beginnenden präzirrhotischen Leberparenchymumbau, jedoch in allen Fällen eine gerade nicht periportale, sondern läppchenzentrale fein- bis grobtropfige sog. hypoxische Leberzellverfettung (Abb. 4), in 2 Fällen begleitet von einer mittelgradigen diffusen Leberzellverfettung. Im Fall 1 waren periportal prominente abgelagerte Gallethromben zu sehen, obwohl makroskopisch keine Cholezystolithiasis gegeben und keine Abflussstörung erkennbar war. Auch konnte eine aszendierte Cholangitis ausgeschlossen werden.

### Histopathologische Befunde im Myokard

**Figure 5:**
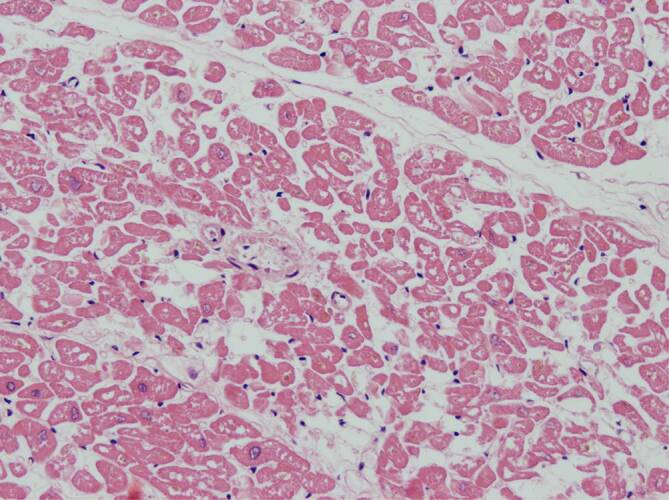

**Figure 6:**
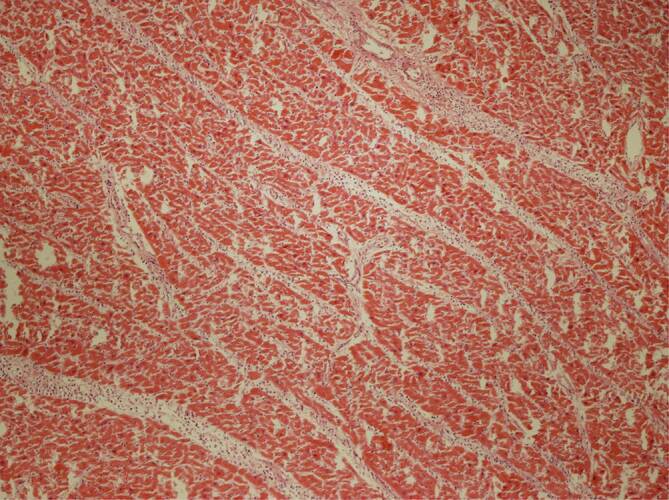

Im Myokard fanden sich jeweils Befunde passend zu einer Myokardhypertrophie mit Kaliberschwankungen der Kardiomyozyten, Kerngrößenunterschieden, interstitieller und perivaskulärer Fibrose, teilweise auch sektorale Koronarinsuffizienzschwielen in Höhe der Spitzen der Stellmuskeln sowie eine fokale Endokardfibrose. Die Kardiomyozyten ließen darüber hinaus, besser erkennbar in quer angeschnittenen Kardiomyozyten, diffus verteilte optisch leere Areale erkennen, bei denen neben einem Verlust an Muskelproteinen auch an eine hypoxische Verfettung von Kardiomyozyten gedacht werden muss (Abb. 5). In einem Fall zeigte sich rechtsventrikulär betont eine streifige interstitielle lymphozytäre Myokarditis (Abb. 6). Der streifige Charakter der Entzündung kann erklärt werden, durch eine sich auch hier entlang der Gefäßwände ausbreitende lymphozytäre Infiltration; ein Bild, welches mit der Annahme einer viralen Endotheliitis und (Peri‑)Vaskulitis gut vereinbar ist.

### Weitere histopathologische Befunde

**Figure 7:**
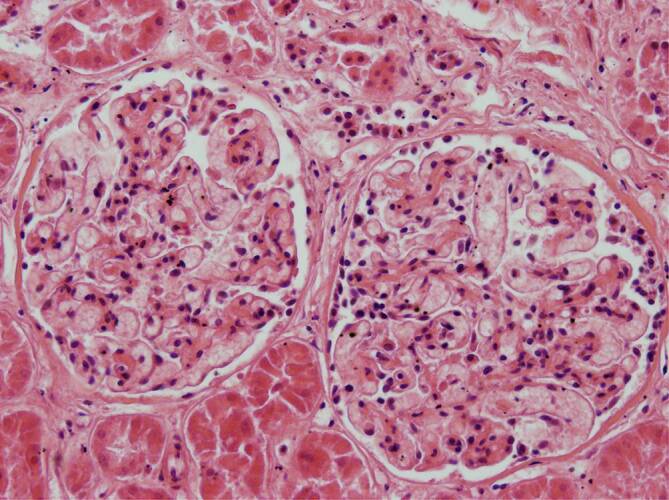

**Figure 8:**
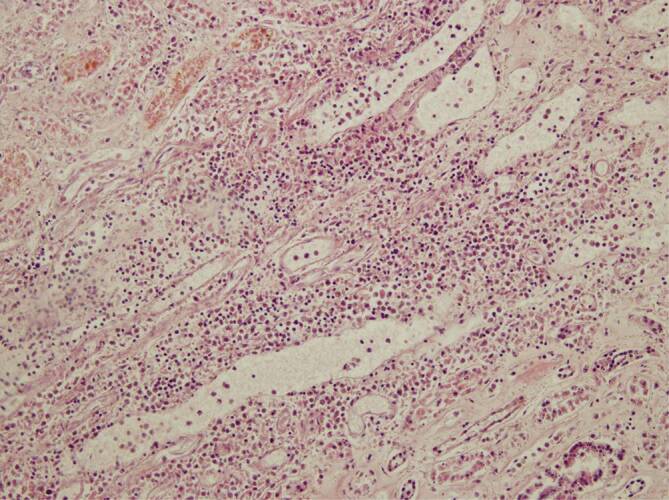

**Figure 9:**
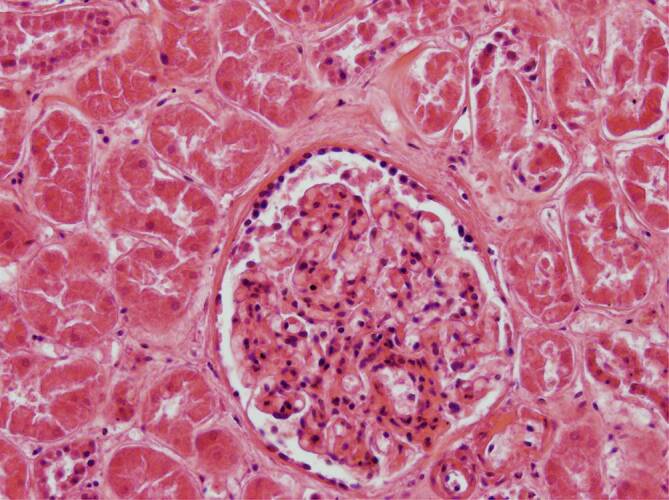

Im Nierengewebe fanden sich in 2 Fällen keine Befunde im Sinne einer Vaskulitis, Endotheliitis oder interstitiellen Nephritis. Auffällig waren jeweils eine deutliche Ausweitung und Ballonierung der Glomerulumschlingen (Abb. 7). Stellenweise zeigte sich eine prominente zelluläre Auskleidung der Bowman-Kapsel (Abb. 8); in einem Fall war eine fokale interstitielle lymphozytäre Nephritis vorhanden (Abb. 9).

**Figure 10:**
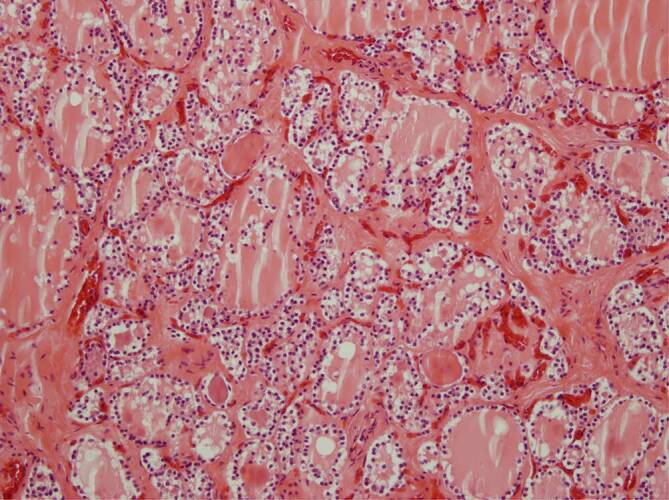

**Figure 11:**
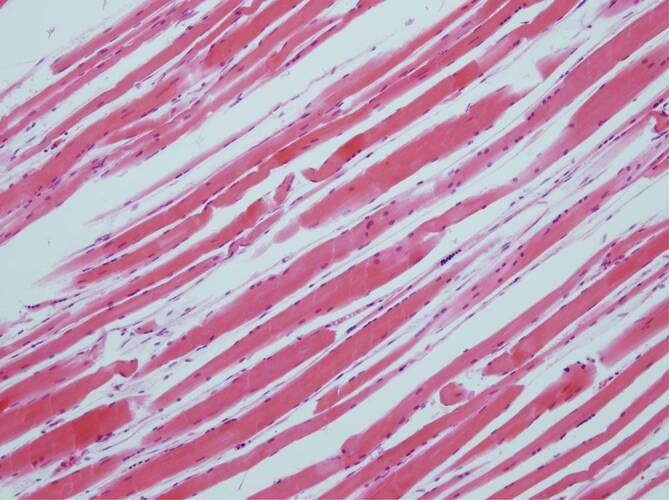

Soweit mikroskopisch untersucht, zeigten die hilären und paraaortalen Lymphknoten bei allen 3 Verstorbenen eine unspezifische chronische Lymphadenitis regionalis. Einzig bei dem nichttherapierten Patienten (Fall 2) waren histologisch im Schilddrüsengewebe vermehrte intrakolloidale Resorptionsvakuolen nachweisbar (Abb. 10). Ein Patient (Fall 3) bot klinisch nach Entwöhnung von der Beatmung und wenige Tage vor seinem Tode das Bild einer „critical illness neuropathy“ and „critical illness myopathy“ mit inkompletter Tetraplegie. Hier zeigten sich histologisch teils schmale, teils aufgehellte Muskelfaserschläuche (Abb. 11).

In allen 3 Fällen waren die hilären und paraaortalen Lymphknoten nicht auffällig vergrößert, auch wenn sich histologisch eine unspezifische Lymphadenitis zeigte. Ebenso fehlte jeweils eine entzündliche Milzreaktion, was jedoch selbst bei ausgedehnter bakterieller Bronchopneumonie nicht zwingend zeitnah zu erwarten ist (Tab. 2).

**Table 2:** 

	Fall 1 – therapiert	Fall 2 – unbehandelt	Fall 3 – therapiert
*Herz*	Myokardhypertrophie, interstitielle und perivaskuläre Fibrose, fokale Endokardfibrose; Zeichen einer Hypoxie		Lymphozytäre Myokarditis
*Lungen*	Peripher betontes Lungenemphysem, interstitielle Fibrose, Alveolitis, ausgedehnte z. T. organisierte hyaline Membranen (Karnifizierung), Curschmann-Spiralen (2-mal), chronische Tracheobronchitis (2-mal), teils chronische, teils floride Tracheobronchitis (einmal), Mikrothromben (einmal)		
*Lymphknoten*	Unspezifische chronische Lymphadenitis		
*Nieren*	Arterio-Aarteriolosklerose, akute Stauungshyperämie, z. T. „ballonierte“ Glomerulumschlingen; prominente zelluläre Auskleidung der Bowman-Kapseln		Interstitielle Nephritis, cholämische Nephrose
*Schilddrüse*	Altersentsprechend unauffällig	Deutlich aktiviert	Altersentsprechend unauffällig
*Leber*	Hypoxische Leberzellverfettung (3-mal); intrahepatische Cholestase (einmal) bei Cholezystolithiasis und chronischer Cholangitis, Portalfibrose, beginnender präzirrhotischer Leberparenchymumbau (einmal)		
*Skelettmuskulatur*	Ohne signifikanten pathologischen Befund		Teils schmale, teils leere Muskelfaserschläuche

### Virologie und Mikrobiologie

Bei zunächst SARS-CoV-2-positiven Befunden in allen 3 Fällen waren die Abstrichuntersuchungen auf SARS-CoV‑2 in den Fällen 1 und 3 in den Tagen vor dem Tode negativ. Autoptisch entnommene Abstriche vom Lungengewebe ergaben folgende Befunde:

- Fall 1: SARS-CoV‑2 negativ; massenhaft *Enterococcus faecalis*,
- Fall 2: SARS-CoV‑2 positiv (Lungen, Herz, Nieren); massenhaft *Proteus mirabilis*,
- Fall 3: SARS-CoV‑2 negativ, jeweils massenhaft *Klebsiella oxytoca, Escherichia coli, Acinetobacter johnsonii, Acinetobacter junii*.

## Diskussion

Es ist bei der Erhebung makroskopischer sowie mikroskopischer pathologisch-anatomischer Befunde unbestritten, dass die Qualität der klinischen Angaben zur Vorgeschichte die Qualität der pathologisch-anatomischen Diagnose und die Interpretation der Histopathologie bestimmt. Klinische Symptome und Befunde müssen mit den postmortal erhobenen Befunden korreliert werden, ggf. sind kausale Zusammenhänge erkennbar, mit Rückschlüssen auf pathophysiologische Prozesse [17].

Klinisch klagen Patienten mit schwerem Verlauf einer SARS-CoV-2-Infektion in der Anfangsphase nicht über Atemnot. Es kann jedoch, nach mehreren Tagen oder auch erst nach mehr als einer Woche, zu einer akuten Verschlimmerung kommen. Werden die Patienten intensivpflichtig, so reicht das therapeutische Spektrum von einer nichtinvasiven Sauerstoffgabe bis zur invasiven Beatmung im künstlichen Koma, teils unter Antibiose, teilweise gefolgt von einem Nierenversagen. Im Einzelfall wird eine extrakorporale Membranoxygenierung (ECMO) durchgeführt.

Eine sich klinisch allmählich entwickelnde Pneumonie und Hypoxie erklärt die hypoxische Leberzellverfettung und die hypoxischen Myokardschäden bei SARS-CoV-2-Patienten mit schwerem Verlauf der Erkrankung. Die histopathologisch darstellbaren, partiell „tapetenartig“ organisierten hyalinen Membranen in den Lungenalveolen machen zusammen mit den weiteren Befunden einer viralen Pneumonie mikroanatomisch nachvollziehbar, dass eine ausreichende Sauerstoffsättigung des Blutes nicht mehr möglich sein kann.

Bisherige Publikationen zur Histopathologie bei SARS-CoV-2-Infektionen betonen Lungenveränderungen, eine Endotheliitis und (Mikro‑)Thrombosen sowie histopathologische Befunde in den Nieren [1, 4, 6, 7, 11, 12, 15, 16, 18].

Histologisch fällt auf, dass sowohl in den Lungen als auch in anderen Organen die lymphozytären Entzündungsinfiltrate eine offenbar gefäßwandassoziierte Ausbreitung zeigen, mit Hinweisen auf eine Endotheliitis sowie auf eine perivaskuläre Entzündung. Die Bindung des Entzündungsinfiltrates an Kapillar- bzw. Gefäßwände auf mikroskopischer Ebene könnte die histopathologisch auffällige streifige Ausdehnung des Entzündungsprozesses erklären, so etwa bei der hier nachgewiesenen SARS-CoV-2-assoziierten Myokarditis (Fall 3). Es bedarf jedoch einer höheren Zahl an Untersuchungen des Myokards zur Klärung der Frage, ob eine histomorphologisch charakteristische, streifige, gefäßwandassoziierte SARS-CoV-2-Myokarditis vorliegen kann.

Bei den 3 hier präsentierten Fällen fand sich jeweils eine Leberzellverfettung vom auch hypoxischen Typ (Abb. 6). Die jedenfalls fokal in den Kardiomyozyten nachweisbaren, vakuolär anmutenden Substanzdefekte (Abb. 7) werden in der Literatur als Folge einer chronischen Hypoxie angesehen [2]. Die Histopathologie lässt insoweit Rückschlüsse auf die Pathophysiologie bei schwereren SARS-CoV-2-Infektionen zu [1, 15, 17]: Die hypoxischen Veränderungen in der Leber und im Myokard sprechen dafür, dass im Rahmen einer SARS-CoV-2-Pneumonie im Zeitverlauf ein zunehmender Teil der Sauerstoffaustauschfläche entzündungsbedingt ausfällt. Das betroffene Lungengewebe zeigt nachfolgend eine unzureichende Ausheilung der Pneumonie mit Karnifikation. Mit Erreichen einer bis zu einem gewissen Grade sicherlich individuell kritischen Grenze, in Abhängigkeit auch von der Vorschädigung des Lungengewebes (Emphysem, interstitielle Fibrose, COPD mit sog. Curschmann-Spiralen etc.), kommt es zu einer mehr oder weniger raschen Abnahme der Sauerstoffsättigung im Blut und in der Folge zur hypoxischen Leberzellverfettung und zur Schädigung der Kardiomyozyten, bei denen, vorbehaltlich immunhistochemischer Untersuchungen des Myokards, neben einer hypoxischen Verfettung auch an einen Verlust intrazytoplasmatischer Muskelproteine gedacht werden muss.

In den Fällen 1 und 3 waren im Lungengewebe Bakterienkolonien nachweisbar und eine auch granulozytäre entzündliche Infiltration, entsprechend führten mikrobiologische Untersuchungen zum Nachweis bakterieller Erreger. Dies entspricht dem auch klinisch bekannten Verlauf einer primär viralen lymphozytären interstitiellen Pneumonie und sekundär bakteriellen Bronchopneumonie mit möglicher Entwicklung einer tödlichen Sepsis [2].

Bei (histopathologisch wie radiologisch darstellbarer) Karnifizierung des Lungengewebes im Sinne einer defekten Ausheilung der Pneumonie wird im Überlebensfall genügend Sauerstoffaustauschfläche erhalten geblieben sein. Je nach Dauer und Intensität der Hypoxie sind jedoch akute wie bleibende Organschäden gegeben. Die Befürchtung ist begründet, dass einerseits die vor der SARS-CoV-2-Infektion bestehende körperliche Leistungsfähigkeit von den betroffenen Patienten nicht wiedererlangt werden kann, und dass andererseits postentzündlich persistierendes karnifiziertes bzw. vernarbtes Lungengewebe mit einer zukünftig erhöhten Anfälligkeit für Pneumonien einhergehen wird. Denkbar ist auch ein erhöhtes Risiko für eine unterschiedlich ausgeprägte persistierende pulmonale Hypertonie mit protrahierter Entwicklung eines Cor pulmonale. Hier bedarf es prospektiver Studien zum postinterventionellen Krankheitsverlauf während und nach Abschluss der Rehabilitation.

Unter den hier präsentierten Kasuistiken konnten autoptisch Thromben nur in einem Fall eines intensivmedizinisch therapierten Patienten nachgewiesen werden. Dies schließt eine pathologische intravasale Koagulation nicht aus. Insofern können weitere z. B. immunhistochemische Untersuchungen zu zusätzlichen Erkenntnissen führen.

Einzig bei dem nichttherapierten Patienten fanden sich in der Schilddrüse deutlich vermehrte intrakolloidale Resorptionsvakuolen als histomorphologisches Äquivalent einer gesteigerten funktionellen Aktivität. Dieser Befund darf als Stressreaktion gewertet werden, die beiden anderen Patienten befanden sich dagegen die meiste Zeit im künstlichen Koma. Eine deutlichere Lipoidentspeicherung der Nebennierenrindenzellen war in keinem Fall nachweisbar.

## Fazit

1. Bei tödlichem Verlauf einer SARS-CoV-2-Infektion dominieren histopathologisch eine interstitielle Pneumonie, eine Alveolitis, dilatierte Kapillaren der Alveolarsepten, partiell organisierte hyaline Membranen, ein partiell proteinreiches Lungenödem und eine Karnifizierung des Lungengewebes.
2. Im Überlebensfall dürften Schäden v. a. des Lungengewebes persistieren, mit zukünftigen gesundheitlichen Risiken: z. B. Leistungseinschränkungen, erhöhtes Risiko einer sekundären bakteriellen Bronchopneumonie nach primär viraler SARS-CoV-2-Pneumonie, Entwicklung einer pulmonalen Hypertonie und eines Cor pulmonale.
3. Die SARS-CoV-2-Patienten können bei akuter Infektion im Verlauf eine allmählich zunehmende Hypoxie mit histopathologisch darstellbaren hypoxischen Schäden (Leber, Myokard) entwickeln (klinisch ARDS).
4. Interstitielle Nephritiden und glomeruläre Erkrankungen können auftreten; eine SARS-CoV-2-assoziierte Myokarditis ist noch nach überlebter Pneumonie möglich.
5. Bei einer SARS-CoV-2-Infektion können zelluläre Dysplasien entstehen, z. B. der Alveolarmakrophagen.
