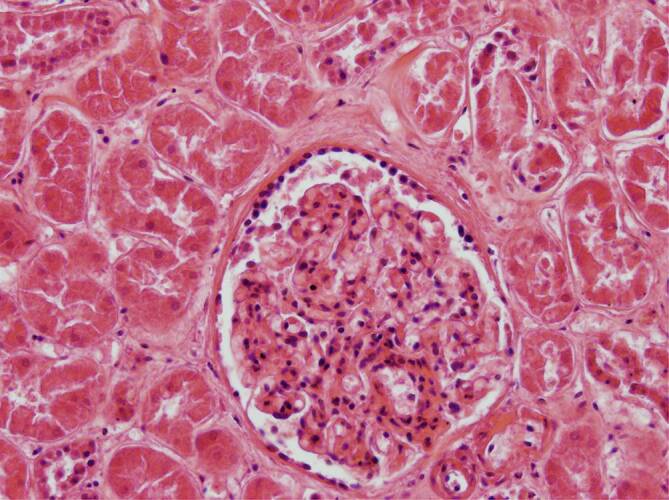# Erratum zu: Histopathologische Befunde bei therapierter und nichttherapierter SARS-CoV-2-Infektion – Bericht über 3 Autopsien

**DOI:** 10.1007/s00194-020-00409-w

**Published:** 2020-09-04

**Authors:** R. Dettmeyer, G. Lasczkowski, A. Weber, T. Wolter, G. Kernbach-Wighton

**Affiliations:** Institut für Rechtsmedizin, Justus-Liebig-Universität Gießen, Frankfurter Str. 58, 35392 Gießen, Deutschland

**Erratum zu:**

**Rechtsmedizin 2020**

10.1007/s00194-020-00408-x

Leider wurden durch ein technisches Versehen die Legenden der Abb. 8 und 9 vertauscht. Wir bitten Sie, die korrigierten Abbildungen zu beachten und den Fehler zu entschuldigen.

**Figure 1:**
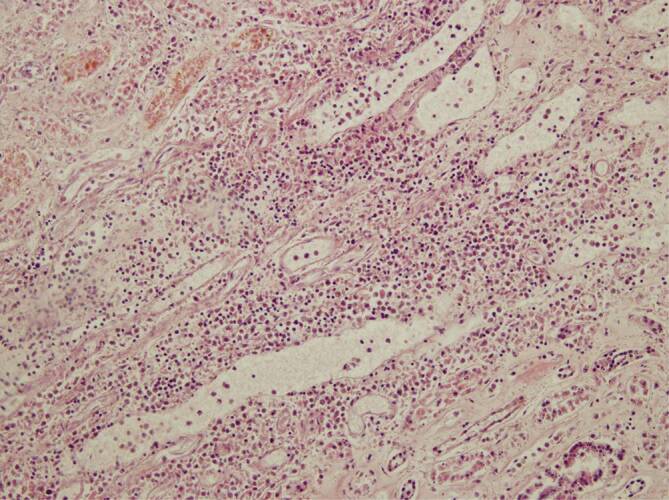

**Figure 2:**